# Review of a specialist Rett syndrome clinic from 2003 to the COVID pandemic: clinic experience and carer perspectives

**DOI:** 10.1186/s13023-024-03483-5

**Published:** 2024-12-20

**Authors:** Emily Sloper, Megan Hunt, Angus John Clarke

**Affiliations:** 1 All Wales Medical Genomics Service, Wales Genomic Health Centre, Cardiff Edge Business Park, Longwood Drive, Whitchurch, Cardiff CF14 7YU Wales, UK; 2 Cardiff University School of Medicine, Institute of Medical Genetics, University Hospital of Wales, Heath Park, Cardiff CF14 4XN Wales, UK

**Keywords:** Rett syndrome, Clinic, Review, Carers, COVID-19 pandemic, Survey, Interviews

## Abstract

**Background:**

We have held a ‘trouble-shooting’ clinic for Rett syndrome patients from 2003 until the COVID pandemic in 2020. The clinic was multidisciplinary, including clinical genetics, paediatric neurology, adult learning disability psychiatry and physiotherapy. Access to specialist communication support and eye-gaze equipment was also often available. We have reviewed the files of patients seen in the clinic and conducted a survey of parents’ and carers’ satisfaction with the clinic and their experiences during COVID.

**Results:**

Of the 117 patients seen in the clinic, records were reviewed of 103 (97 female, six male) who attended a total of 123 appointments. The records were unavailable for 14 patients. The most common reasons for referral were assessment of ‘episodes’ of uncertain nature (possibly epileptic, possibly autonomic), the wish for a general review by an experienced team, and questions about the diagnosis. We discuss the nature of the advice we were able to provide and offer some brief case vignettes. We wrote to the parents or carers of all patients seen and 63 respondents were willing to be interviewed about the clinic and their experiences during COVID. Respondents were generally complimentary about the clinic team, emphasising the value of a specialist clinic for those affected by a rare condition. Respondents gave insight into the range of problems experienced during COVID, especially the isolation resulting from the withdrawal of services, demonstrating the value of community support. Some respondents mentioned the shift to remote consultations, which they hoped would continue after COVID for its convenience. However, others talked about how difficult it is in a remote consultation to explain the problems of the affected family member to professionals who do not know the patient or know about Rett syndrome.

**Conclusions:**

Our findings demonstrate the value of a disease-specific clinic provided by staff experienced with the particular rare condition. Meeting the needs of patients with ultra-rare conditions presents additional challenges. We have also found that the shift to holding a virtual clinic during COVID brought the benefit of convenience but was unsatisfactory in other ways, as it makes clinical assessment more difficult and fails to overcome the sense of isolation during a pandemic.

## Background

### Clinical services for rare conditions

The role of specialist clinical services for those affected by rare conditions is complex and can be difficult to define. It will vary with the demographics and geography of a country [15] and its system for the funding and provision of healthcare. For the less rare conditions, such as cystic fibrosis (CF) and Duchenne muscular dystrophy, clinics organised on a regional basis may be able to provide much or all of the specialist care required, if they coordinate effectively with local and community services, such as physiotherapy. Expert review of individuals with some rare conditions is known to contribute positively to their management [31] as well as patient and carer satisfaction and wellbeing [26, 34]. Individuals who are unable to access coordinated, specialist care for their rare condition are reported to experience delays in diagnosis, suboptimal management of their condition and a negative impact on their emotional and psychological wellbeing [26].

For some rare conditions requiring complex, multidisciplinary care, the organisation of a national clinic may be appropriate [31]. However, for many of the numerous but very rare conditions we are considering, such provision of expert care would be a major challenge. Indeed, the ability for clinicians to acquire sufficient experience of supporting patients with many such conditions is limited, especially for ultra-rare conditions (affecting fewer than 1 in 50,000). Furthermore, many of a patient’s needs may be common to those affected by other neurodevelopmental disorders. For such conditions, a different model of care has to be found. While paediatric services may be organised around the One Child framework and provide an integrated service [2], this approach may be less widespread in adult services, so difficulties may arise for the teenager transitioning to adult services [35].

One approach, where a centre has some experience with a condition, is for the patient’s local clinicians to provide the hands-on care, but for the expert centre to offer occasional, perhaps annual, ‘patient reviews’, as well as back-up support or advice by trouble-shooting for the local team. However, even with this model, there can be difficulties. Experience with a condition may have been built up by one clinician through their research, so that the centre’s expertise may be based largely on the experience of one or two clinicians. The centre may then accumulate a collective experience over time, but only if the volume of patients seen is sufficient. A specific problem that arises in some conditions where there is vulnerability to infection, is the need to prevent cross-infection between patients; this may drive the pattern of service provision, as with cystic fibrosis. For many neurodevelopmental conditions, it is vital that countries provide a dispersed system of therapy centres—such as the Children’s Centres found in the UK, but provided for adults too—to make available in each locality the range of resources (assessments and treatments) that will be required by patients with many different rare conditions.

### Rett syndrome (RTT)

Rett syndrome (RTT) is a neurodevelopmental condition which is typically defined by ‘apparently normal’ development for 6 months or more, and then stagnation followed by a regression of development—typically in the second year of life—that includes a loss of social contact, of purposeful hand movements and other skills and the development of hand stereotypies [16, 23, 24]. The regression distresses both the child and the family. Development then stabilizes and there is often some re-emergence of social contact and some other abilities, although leaving the child with usually profound problems with autonomic function, cognition, communication and movement [4, 12, 17, 33]. RTT mostly affects females and accounts for some 10% of profound neurodevelopmental problems in girls. It is a rare condition but sufficiently common (about one in 12,000 female births) that clinicians can acquire clinical experience of supporting patients and families and apply this learning to other patients. As such, it has been argued that there is a clear need for specialist clinics for individuals with Rett syndrome [14]. There are useful guidelines for the monitoring and medical management of patients with RTT [7] and for the management of specific problems often found in RTT [6, 13, 14].

The usual cause of RTT is a pathogenic variant in *MECP2*, a gene on the X chromosome [1, 3]. The elucidation of the usual molecular basis of RTT [28] has stimulated efforts to develop effective treatments [11, 18, 21, 25]. As yet, there remains no cure, although clinical trials of gene-based therapies are in progress. Precisely what would constitute a “cure” for RTT is open to some debate, especially for older children or adults [5, 32].

Individuals with Rett syndrome have increased rates of mortality and morbidity when compared to individuals without Rett syndrome. They typically experience neurodevelopmental difficulties, autonomic dysfunction, seizures, scoliosis, difficulties with feeding and with nutrition and growth, bone density, mood and behaviour, and sleep, as well as their general health [17, 22, 33]. Distinguishing epileptic seizures from autonomic disturbance may be difficult and lead to the over-diagnosis of epilepsy and over-treatment with anticonvulsants [8, 9], which then impacts arousal, cognition and bone health, leading to additional adverse consequences. While about 90% of patients with RTT will have epileptic seizures at some stage in their lives, the prevalence of epilepsy is less than half that [27]. Difficulties with communication and other neurological manifestations may complicate the clinical picture of general health conditions and make the diagnosis of comorbidities more difficult.

### The Cardiff Rett syndrome clinic

The Cardiff Rett clinic has operated since 2003 as one of the few UK services focused on this condition. It was established in 2003 with support from Dr Alison Kerr (who established and maintained the British Isles Rett Syndrome Survey) and the family support organization, Rett UK (formerly Rett Syndrome Association UK). This multidisciplinary clinic brought together a functioning team of clinicians, who already had specialist knowledge of Rett, from 2003 until the coronavirus pandemic in early 2020, when the clinic was interrupted because of the vulnerability of RTT patients to infection, because clinic space was taken over by laboratory staff to enable social distancing, and because of staff retirement. This break in provision has provided a natural opportunity to review our experience.

The clinic has always been patient- and carer-led, responding to concerns or queries identified by the carers, and triggered by the patient’s local clinical team. The intention of the clinic is particularly to assist with referrals relating to questions about a patient’s diagnosis (e.g. whether a patient has RTT or not), questions from the families or carers of a newly diagnosed patient (such as, “What does the future hold for my child?”), advice about the practical management of specific problems encountered by the patient, advice about the transition to adult services, or a general review when a patient had not seen specialists for a lengthy period. The clinic was established as an occasional, trouble-shooting clinic and not as the site of regular follow-up or general coordination of a package of care, and those referred came from too wide an area for that to be feasible.

Attendance at the clinic has typically comprised a joint medical appointment with three specialists (a clinical geneticist, a paediatric neurologist and a learning disability psychiatrist), an appointment with a specialist physiotherapist, and often also other specialist assessments, for example, with a speech therapist or communication specialist. Sometimes patients were offered a trial of eye-gaze technology, when the clinic was attended by a representative of Tobii, one of the commercial providers of eye-gaze technology.

The aim of this paper is to report the experience of the Cardiff Rett syndrome clinic and to give the perspective of the carers who attended the clinic. This was to identify the reasons for referral to the clinic, give the carers’ opinions about its usefulness, and to draw conclusions that might be helpful to other centres wishing to set up a comparable service.

## Methods

Patients could be referred to the clinic if they had a confirmed or suspected diagnosis of Rett syndrome or a Rett-like disorder and their primary clinician and/or carers thought they would benefit from specialist input. *MECP2* pathology, being neither necessary nor sufficient for a molecular diagnosis of RTT [16], was not required for referral. Patients and/or carers were encouraged to obtain a formal referral from their clinician to the clinic to ensure that background clinical information was provided, although this was not mandatory: carers could self-refer directly if they wished. Referrals were also occasionally received from within the clinic team, when a multidisciplinary team approach was thought to be appropriate. Referrals were accepted for patients from the NHS or overseas. Referrals from beyond NHS Wales required health authority approval (if the patient was referred from within the UK NHS) or a formal funding agreement. The patients and/or carers were encouraged to travel to Cardiff for face-to-face clinic appointments. Follow-up consultations were offered to a few patients by telephone but these were not included in the data. These telephone appointments were usually brief and were arranged to answer specific questions raised by families or carers. Virtual consultations were not offered until the COVID pandemic and are not included in the clinic review data or the questionnaire study.

The practitioners at the clinic included a Consultant Geneticist, Consultant Paediatric Neurologist, Consultant Learning Disability Psychiatrist and Physiotherapist. A family support worker from Rett UK was usually present. Other personnel who attended when possible included eye-gaze technicians and speech/communication therapists. Trainees in clinical genetics, paediatric neurology and physiotherapy sometimes also attended.

There were two main elements to this review of the RTT clinic. First was a retrospective case note review by the clinical team (AC, ES). Second was a medical student project that involved contacting families to obtain feedback from carers (MH). The former element was classified as a service evaluation so that, under NHS rules, it did not require research ethics review. The latter element was also classified as a service evaluation and approved as a student project through Cardiff University School of Medicine.

A retrospective case note review was performed for all patients with Rett syndrome who attended the Cardiff Rett clinic from 2003 to late 2019. Patients, female and male, were included if they had a clinical diagnosis of RTT or atypical RTT [16]. Patients were excluded if their records were unavailable. Information from the patient records was collated, to include patient demographics, genetic test results and reasons for their referral. The data were compiled in an anonymous format.

In addition, in 2021 a letter was sent to the carers of patients who had ever attended the clinic, inviting them to participate in an interview with a Cardiff University medical student to discuss their experience of the clinic and their experiences of living with RTT during the COVID pandemic. Where there was no response to the letter or our records showed no valid address, and the family lived in the UK, we checked the patient’s or family’s contact details with their General Practitioner and, where we had a record of an email address or telephone number, we attempted a single contact using those methods. When there was no response to these modes of contact, we did not persist with further attempts.

Interviews were held over a period of two months, in June and July 2021. Patients and carers were asked a series of questions via telephone and/or email to evaluate their experience of the clinic, the perceived strengths and weaknesses of the clinic, and their experiences of living through the pandemic (see the Appendix for the list of questions). Responses were qualitative using free text as well as quantitative using a 5-point Likert scale.

## Results

### Case note review

117 patients were recorded as having attended the Cardiff Rett clinic between 2003 and 2019. One clinic set up in early 2020 had to be cancelled because of the Covid-19 pandemic. Records were unavailable for 14 patients. The records were reviewed for the remaining 103 patients.

#### Clinic attendance

Typically, 4–6 patients attended each clinic. Clinics were held 2–3 times per year in the University Hospital of Wales, Cardiff. Data was reviewed from a total of 123 clinic appointments: 83 patients attended the clinic once, 11 attended twice, two attended three times and three attended four times; data was incomplete for four patients. Those patients who attended multiple times had usually been re-referred by their local clinician and were often local patients who could travel more easily.

#### Demographics

97 of the 103 patients (94.2%) were female, with 6 patients being male. The patients’ ages at their first clinic attendance ranged from 1.93 years to 56.4 years old (Table 1). 26 patients were from Wales, with the 71 others travelling from elsewhere in the UK or abroad.

**Table 1:** Demographics of the patients who attended the Rett clinic

Demographic	Number of patients	%
*Gender*		
Female	97	94.1
Male	6	5.9
*Age at first clinic attendance*		
0–9 years	46	44.7
10–19 years	22	21.3
20–29 years	18	17.5
30–39 years	8	7.8
40–49 years	4	3.9
50–59 years	4	3.9
Incomplete data	1	1.0
*Number of clinic attendances*		
1	83	80.1
2	11	10.7
3	2	1.9
4	3	2.9
Incomplete data	4	3.9

#### Variant analysis

Of the 97 female patients, 58 (60%) had confirmed *MECP2* variants. Of the female patients who did not have confirmed variants in *MECP2*, one had a chromosome Xp22.2 deletion and three had *CDKL5* variants. The remainder did not have genetic testing or had negative mutation analysis for *MECP2*.

Of the six male patients, none had *MECP2* variants and one had a confirmed *ARX* variant. The remaining five did not have a known molecular diagnosis at the time of their clinic appointment.

#### Reasons for attending

**Fig. 1:**
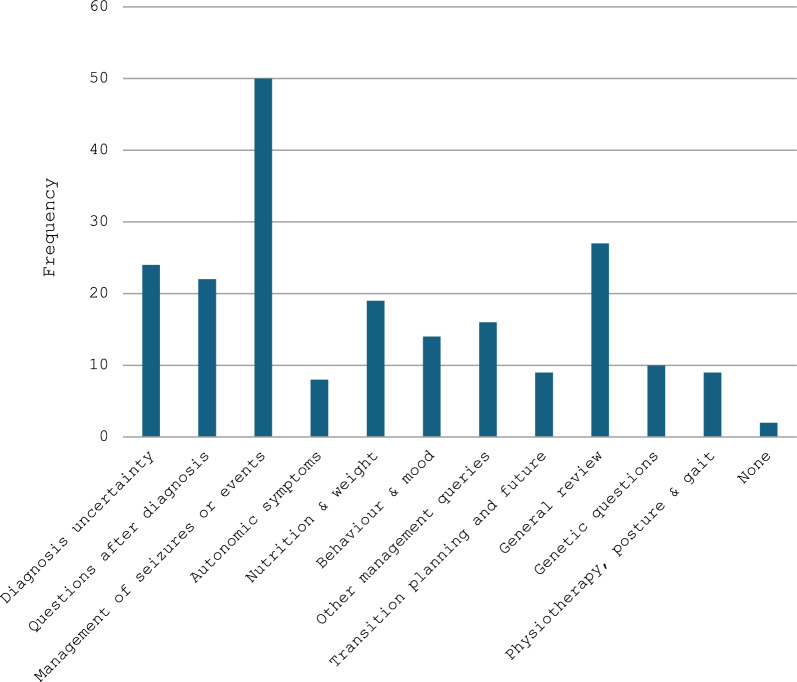
Documented reasons for attendance at the Rett clinic

There were 123 clinic appointments in total. 79% of patients had more than one documented reason for their referral and attendance at the clinic (Fig. 1).

The most commonly documented reason for attending the clinic was for the management of seizures or seizure-like ‘events’, which were discussed in 50 (41%) of the appointments. 27 (22%) of the appointments included a general symptom review, 24 (20%) of the appointments included discussions around uncertainty of the patient’s underlying diagnosis and 22 (18%) included discussions about the patient’s recent diagnosis of Rett or Rett-like syndrome, including their prognosis and management.

Other, less common reasons for appointments included advice about nutrition, weight and diet, autonomic symptoms, mood and behaviour, the interpretation of genetic test results and the possible risk of recurrence in the family. Several referrers also sought advice regarding posture and scoliosis or a general deterioration in symptoms. Nine families or carers also wished to discuss transition to adult care and/or the long-term prognosis. For two appointments, the rationale or intended purpose for the referral and appointment was not recorded.

### Parents’ and carers’ perspectives

**Fig. 2:**
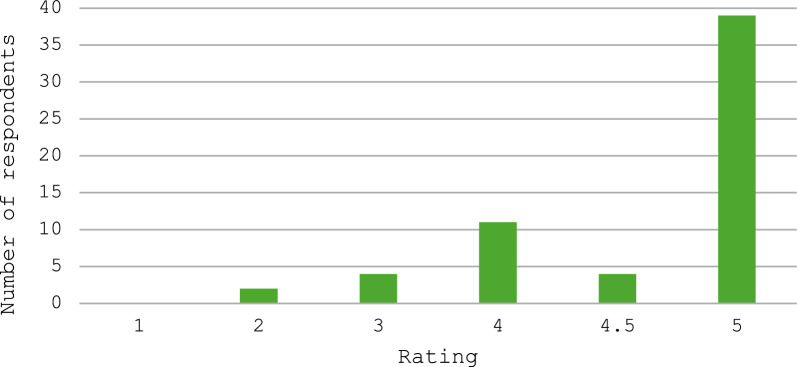
How respondents rated the Cardiff Rett clinic as a service. Respondents rated the service on scale from 1 to 5, where 1 was very bad/awful, 4 was good and 5 was excellent/very good

**Fig. 3:**
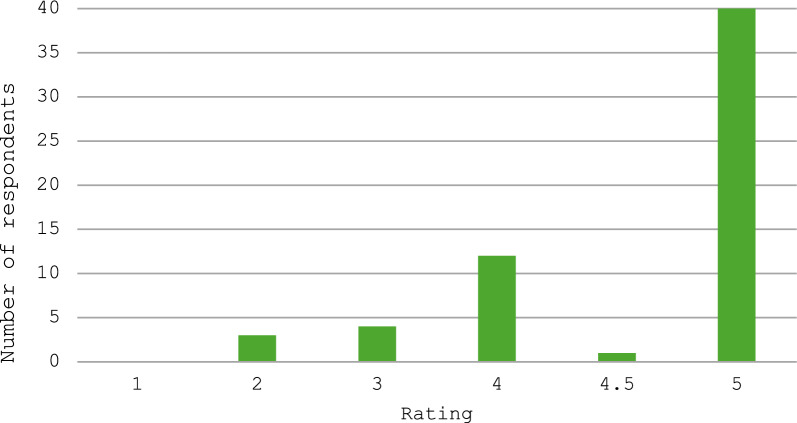
How respondents rated the Rett clinic in terms of how helpful it was for their child and their child’s care. Respondents rated this on a scale from 1 to 5, where 1 was very bad/awful, 4 was good and 5 was excellent/very good

There were 63 respondents to the questionnaire, of whom 61 gave responses about the clinic and 54 gave responses about the impact of COVID-19. Parents and carers generally rated the clinic highly, with 54 of the 61 respondents giving the clinic an overall score of at least 4 out of 5, where a score of 4 is ‘good’ and a score of 5 is ‘excellent’ (Table 2 and Fig. 2). We also recorded data separately for perceptions of the impact of the clinic specifically on the care of the affected person and found a similar pattern of responses, with 53 rating it 4 or higher (Table 2 and Fig. 3). Both questions show a similar spread of answer data, with similar shape of graph in Figs. 2 and 3.

**Table 2:** Response data from questionnaire on parent/carer perspective on the Rett clinic

Question	Response	Response frequency
Was the clinic helpful?	Yes	55
	No	3
	Other	3
In general, what did you think about the Rett Clinic as a service? How would you rate it on a scale 1–5?	1	0
	2	2
	3	4
	4	11
	4.5	4
	5	39
How important/helpful/useful was the service to your daughter in particular? How would you rate it on a scale 1–5?	1	0
	2	3
	3	4
	4	12
	4.5	1
	5	40

#### Impact on patient care

Families were asked for specific feedback about the clinic. Fifty-five respondents found the clinic helpful, and only three respondents said the clinic was not helpful. Twenty-four families praised the level of expertise/specialist knowledge available at the clinic and highlighted the value of having so many specialist clinicians together to review their child in one room. Fifteen respondents commented specifically on the value of seeing a specialist physiotherapist, who was able to help with problems relating to posturing, scoliosis, constipation, and abdominal distension. Positive comments were also received about the attendance of other specialists, including access to advice from the neurologist, an epilepsy specialist, who helped several families to achieve control of previously refractory epilepsy. Thirteen families mentioned the value of the chance to trial eye-gaze technology for the first time and many have gone on to use this technology to aid their child’s communication.

Eleven respondents commented that the clinic had been useful in terms of accessing specific services or accessing appropriate support for their child at home, whether by specific referral or written statement from the specialists, through getting a confirmed diagnosis, or by knowledge gained on what support is available and recommended for their child. One family particularly valued the confidence the clinic gave them to speak up for their daughter’s needs in the community. Many parents spoke about the frustration they experienced from being the ‘expert’ on RTT when accessing healthcare in their locality. In particular, eleven respondents mentioned how nice it was to speak with people who were familiar with and interested in Rett’s, and several commented about not having to explain the condition. Eleven families also found the clinic helped confirm their diagnosis or gave a specific label to their child’s condition, which had benefits including parent relief, parent confidence and accessing appropriate support.

Other recurring comments included how friendly and welcoming the clinic staff were, how informative the clinic was, especially for those with a new diagnosis, and how it gave families an idea of what to expect for the future and what the ‘normal’ might be for a child with RTT. Parents/carers also valued having the chance to meet other individuals or carers of individuals with RTT.

#### Selected case vignettes

As illustrated above, there were many common themes in reasons for the referrals of the patients and their families. Two commonly seen concerns are well represented by one case. The parents of a young, recently-diagnosed child asked us how to help her get the most out of life. They then returned, a few years later, to say that her identified needs were not being met and to ask how we could support them in achieving this. This has been an ever-increasing cry from the heart of the families of disabled people over this last decade or more of austerity.

With more specific clinical questions, narrower and more technical responses were provided. One 10-year-old patient was having episodes of pain, respiratory dysrhythmias and seizures. She had already had an arthrogram of the hips, an abdominal ultrasound scan, studies of oesophageal pH, tests for *H. pylori,* urine microscopy and culture and a dental examination. We suggested that she should be withdrawn from topiramate and that attention be paid to nutrition, vitamin D status and bone health including measurement of vitamin D and PTH levels.

A 21-year-old presented with a history of weight loss and emotional outbursts over a few months. This followed the death of her grandmother. From the context, and the lack of indications of a specific medical problem, we thought that a bereavement response was possible. We suggested that her family and carers could consider developing ways to remember her grandmother with her.

One 9-year-old girl presented with two problems: long-standing self-injury and a sudden recent deterioration. We suggested a focus on distraction rather than restraint for the self-injury, although recognizing that gentle restraint will sometimes have a place. Within the constraints of the clinic, we could give little advice about the deterioration. It could have resulted from an exacerbation of seizures, a late episode of RTT-related regression, a response to pain of undetermined origin, or even a response to a recent (and probably unrelated) immunisation. We suggested that the referring clinicians continue to act on any suspicions as to possible medical causes of deterioration, although their investigations—and our brief assessment in clinic—had provided no specific clinical pointers.

Other specific questions often asked in the clinic centred on the genetics of RTT and questions of reproductive risk. For example, families wishing to understand the chance of a healthy older sister ‘carrying’ the affected girl’s pathogenic variant in *MECP2* and discussions about germline mosaicism.

Other genetic questions were more complex. For example, questions about whether exon-skipping would work as therapeutic strategy for their daughter’s specific *MECP2* variant, or how would one separate the effects of a non-RTT *MECP2* variant from their child’s likely pathogenic exon 4 rearrangement.

Our final vignette concerns the question of resuscitation for a girl with RTT, if she were to suffer a cardio-respiratory arrest. She had been having difficult episodes of an uncertain nature that were not being managed very successfully. Her parents were distressed to find that the hospital had added a ‘Do Not Resuscitate’ notice to her charts, when she was an inpatient. This had been done with the involvement of neither the parents nor the Court of Protection and would therefore probably not have been valid. We recommended that communication—a process of discussion between the parents and the hospital team—should be the first step. It is all too easy to see how the family could lose confidence in a medical team if decisions are imposed without an opportunity for them to mould any decision that might be reached, quite apart from any question of its legal validity. A loss of confidence in the professional ethos of a medical team can easily lead to serious conflict that helps no-one, especially not the child patient, and can leave the parents with a bitterness for years to come.

#### Possible improvements to patient care

Nineteen respondents made comments pertaining to the difficulty of getting a clinic appointment, either commenting on the length of time they waited for an appointment, difficulty getting an appointment or the infrequency of appointments. Many suggested improvements such as increasing the frequency of clinics, for example having clinics annually or once every two years, and improving follow-up, such as with telephone/zoom appointments or a dedicated service for families to phone with problems or queries.

Fifteen respondents commented on the distance required to travel to the clinic, wishing that it were nearer. Many felt that it would be too far and too difficult to travel there again and suggested either more locally based clinics or use of telehealth methods to facilitate easier appointments. Forty-three respondents felt that they would not suggest any improvements for the clinic service. Other individual improvement suggestions included running an age-related clinic for older individuals with RTT, consider translating information resources into other languages, including more specialists, advising on transition from child to adult services, and providing more opportunity to meet other families/parents.

#### Management suggestions

As part of the clinic, suggestions were often made to the referring doctors regarding the management of patients with RTT. Examples of the more common management suggestions are detailed below.

1. Monitor weight in adults as well as children. Weight can be high as well as low and it is important to recognise that the target weight in RTT is usually less than in others of the same age and height.
2. Disentangle different types of episodes if possible. This may require video-recording, EEG, ECG and respiratory monitoring, and sometimes transcutaneous blood gas monitoring and sleep studies.
3. Be slow to make changes in epilepsy management. Do not increase anti-epileptic drugs to treat non-epileptic events and do not medicate EEG changes alone.
4. Be aware of depression as a possible cause of deterioration but ensure physical health concerns are fully addressed before prescribing anti-depressant drugs.
5. It is generally helpful to have molecular genetic confirmation as to the cause of a condition that attracts the clinical diagnosis of RTT.
6. The needs of a child with RTT are very different from those of a child with autism, especially their social and schooling needs.
7. Encourage older parents, who are coping well in providing care for their affected (now adult) daughter to engage with social services.
8. When managing anorexia, consider the effects of any medication and consider a change. If that does not help, assess for the possibility of gastro-oesophageal reflux, *H. pylori* infection, malnutrition, depression or respiratory dysfunction.
9. For problematic drooling, try hyoscine patches then oral medication. If severe and persistent, consider an ENT referral for salivary gland duct diversion or Botox injection.
10. Remember to signpost families to the Community Learning Disability Team (applicable for patients in the UK).
11. Remember preventive management of bone health. This includes discontinuing anti-epileptic drugs when they are not required; giving calcium and vitamin D supplements; encouraging walking and/or weight-bearing exercise; encouraging outdoor activities for sunlight exposure.
12. Physiotherapy is essential to maintain mobility when possible and to optimise the posture and reduce the risk of scoliosis. Horse riding and music therapy may have a role here too.
13. Remember that oesophageal reflux has multiple presentations as it can cause pain, anorexia and ‘episodes’.

#### The impact of COVID-19

The respondents indicated that the impact of the first 15 months of the COVID-19 pandemic had been massive, with many families feeling “abandoned” and “neglected” by services and “forgotten about”. The closure of day care services, respite care and schools/colleges hugely increased the care burden on families. Access to healthcare varied depending on the familiarity of local healthcare teams with the family. Some families described excellent support, with understanding general practitioners (GPs), people (pharmacy staff and volunteers) willing to deliver prescriptions, specialists being “at the end of the phone” and supportive community teams. Others however, faced many difficulties, such as trying to explain problems over the phone to a GP with no understanding of RTT, having to fight to accompany their daughter to A&E, or even just struggling to get any form of appointment or telephone conversation with a doctor.

Many (14) families commented on the negative impact of the closure of sports and activity services such as hydrotherapy, swimming and physiotherapy. This had negative effects on mobility and joint health for some girls, who relied on these services; for others, it had a negative effect on emotional wellbeing. Further to this, at least 19 families commented on the social impact of COVID-19 on their child. Many were unable to see friends or family and were socially isolated but with little understanding of why. Several families noted behavioural changes and considered their daughters might be depressed.

One of the most prominent changes to service provision was the use of phone/video calls and other technology in place of face-to-face appointments and consultations. Twenty-one families commented on the delivery of consultations via phone/video and twelve of those noted it as a positive change they would like to see in the future, stating benefits such as reduced stress and travel time, increased convenience and reduced disruption to daily life.

## Discussion

Rett syndrome is a complex condition which requires multi-disciplinary, specialist management in a suitable environment to optimise patient and family experience and, ultimately, their support and care. The Cardiff Rett clinic aimed to promote this by offering a specialist clinic service to patients or family members who required additional support for the condition beyond that offered through their local clinical services.

As would be expected for a specialist RTT clinic, the majority of patients who attended were female and the majority had a *MECP2* variant. Most patients attended the clinic only once, perhaps reflecting the difficulties of travel to the clinic, its specialist nature, and their satisfaction with their usual care.

It is known that patients with rare conditions frequently attend multiple clinic appointments with different teams or clinicians in a variety of settings and that this can result in poor communication between specialities and uncoordinated care [29, 34]. The feedback for the Cardiff RTT ‘trouble-shooting’ clinic demonstrated the positive impact that a specialist multidisciplinary team can have on the patients, carers and families. For example, they valued the input they received from the neurologist for the management of refractory epilepsy and the specialist physiotherapist for advice on specific concerns, both of which may been more difficult to access locally. Families also valued the ability to trial eye-gaze technology, with many not having had an opportunity to trial this previously.

The main documented reasons for clinic attendance included advice on seizure management, as well as general symptom advice. In many cases, the clinic served in a problem-solving capacity, such as by addressing the management of refractory epilepsy and seizure-like events in nearly half of all appointments. The need for discussion about epilepsy management is perhaps unsurprising, given that some 80–90% of individuals with Rett syndrome are reported to have epilepsy at some stage in their lives although the prevalence of epilepsy is usually about half that, so that epilepsy and its treatment both contribute significantly to the impact of the condition [10, 20, 27]. The difficulty in distinguishing epilepsy from autonomic disturbance adds to the clinical challenges.

Discussions around a patient’s diagnosis of RTT and information giving were also commonly reported components of the clinic appointments. Families and carers concurred with this, stating that they appreciated the opportunity to discuss their child’s diagnosis, prognosis and future care, highlighting the importance of having an opportunity to attend a specialist service after the diagnosis of a complex rare condition. Of note, caregivers particularly highlighted the value of seeing clinicians who were familiar with their child’s rare diagnosis. Specifically, six caregivers commented on the importance of not having to explain the diagnosis to clinicians and many spoke of the frustration they experience of continually being needed to become an ‘expert’ on RTT. The usual role of this clinic as a once-off, trouble-shooting clinic will explain the differences between the reasons for referral to our clinic and the spread of more general caregiver concerns found by Neul et al. [19].

In order to improve the clinic further, an increased number of clinics could be beneficial to families and follow-up opportunities could be optimised. In an increasingly digital era, there would also be the potential to benefit from virtual follow-up appointments [30].

Our interviews with carers during the COVID-19 pandemic reflected the circumstances of the times. The reports of services breaking down and many families feeling abandoned were harrowing and had a profound impact on the quality of life for the patients and those around them.

## Conclusion

The Cardiff Rett clinic provides an example of a highly specialised service providing significant benefit to the affected individuals and their families through the provision of information, specialist advice and management recommendations. A collaborative multidisciplinary approach, together with availability of information and support for patients and their carers, has been key to the success of the clinic. The challenges of providing quality care during the COVID-19 pandemic led to the closure of the clinic and, this reflected a more general deterioration in the provision of care in the community that led to many families struggling to cope and often feeling abandoned. It will be important for rare disease clinical services, as well as patients, families and advocacy organisations, to respond to the challenges set out in the UK Government’s UK Rare Diseases Framework [30] including more rapid diagnosis, increased awareness of rare diseases among health professionals, better coordination of care, and better access to specialist treatments. Furthermore, these practitioner groups should reflect on their experiences during the pandemic and ensure a better planned response to comparable challenges in the event of another such pandemic.

Meeting the needs of patients with ultra-rare diseases will provide additional challenges as few clinicians will have much experience of such conditions. It may be helpful to develop a model of services with flexible provision of community-based care with ready access to specialists experienced in providing care for such patients as a group. This will require specific funding to achieve better planning and staff training to meet the needs that such patients often have in common, despite their conditions being distinct.

## Limitations

The authors acknowledge several limitations to the study, including that it was conducted as a service evaluation rather than being designed as research to generate new knowledge. Two significant limitations were the retrospective nature of the survey, that relied on the quality of the case notes and clinic letters, and the inability to include all individuals who attended the clinic in the interview process. There was no funding available for the study and, therefore, follow-up data was not available. This could be the focus of further work in the future.

## Data Availability

The clinical data that support the findings of this study are not openly available due to reasons of sensitivity and patient confidentiality but de-identified data are available from the corresponding author upon reasonable request. Access to pooled anonymous clinical data could be provided, and access to de-identified survey and questionnaire results could also be provided. Data are located in controlled access data storage at Cardiff University.

## Rett clinic questionnaire used as basis for remote interview with parents or carers

### Questions Regarding Cardiff Rett syndrome clinic

- Did you find having a tertiary clinic (i.e. the Cardiff Rett Syndrome Clinic) helpful?
- In what ways has it been useful/not useful?
- What were the strengths and weaknesses of the service?
- How could the service be improved?
- In general, what did you think about the Rett Clinic as a service? How would you rate it on a scale 1–5? (1 being very bad/ 5 being very good)
- How important/helpful/useful was the service to your daughter in particular? How would you rate it on a scale 1–5? (1 being not helpful at all/ 5 being very helpful)

## Questions regarding the impact of COVID 19

- What impact has COVID had on your access to medical care, social support and education?
- Have these services changed during COVID times?
- What problems have you encountered in terms of healthcare, education and social support during COVID?
- Were the solutions put in place during COVID effective?
- Are there any changes that have occurred during COVID that you would want to see continue or be developed going forward?

## Abbreviations

COVID-19: Coronavirus disease 2019
ECG: Electrocardiograph
EEG: Electroencephalograph
ENT: Ear, nose and throat (i.e. otorhinolaryngology)
*MECP2*: (The gene for) Methyl-CpG-binding protein 2
NHS: National Health Service
RTT: Rett syndrome
UK: United Kingdom
